# Special Issue “State-of-the-Art Polymer Science and Technology in Greece”

**DOI:** 10.3390/polym15051264

**Published:** 2023-03-02

**Authors:** Stergios Pispas, Spiros H. Anastasiadis, Hermis Iatrou

**Affiliations:** 1 Theoretical and Physical Chemistry Institute, National Hellenic Research Foundation, 48 Vassileos Constantinou Ave., 11635 Athens, Greece; 2 Institute of Electronic Structure and Laser, Foundation for Research and Technology-Hellas, N. Plastira 100, 70013 Heraklion Crete, Greece; 3 Department of Chemistry, University of Crete, Vasilika Vouton, 71003 Heraklion Crete, Greece; 4 Department of Chemistry, University of Athens, Panepistimiopolis, Zografou, 15771 Athens, Greece

Polymer science and technology is an active and continuously developing field of research and innovation in Greece. Many research groups in the country are active in different subfields of polymer science and technology including polymer synthesis, polymer characterization and properties elucidation, polymer physics, polymer physical chemistry, and the theoretical description and understanding of polymer systems. 

The aim of this Special Issue has been to collect original contributions, both research papers and reviews, demonstrating recent results and advances from the polymer-related research performed in Greece at the beginning of the 21st century. A brief account of the works presented in the issue is given below.

Iatrou and coworkers report on the synthesis of a novel monomer, namely N^ε^-9-fluorenylmethoxycarbonyl-l-lysine N-carboxy anhydride (N^ε^-Fmoc-l-Lysine NCA). The monomer was used for the synthesis of linear peptide homopolymers and well-defined block-graft lysine and histidine-based copolypeptides [1]. The new copolypeptides were characterized in aqueous media in terms of their conformational and aggregation properties under different conditions. The future perspectives of using this monomer for the synthesis of copolypeptides of complex macromolecular architecture, able to be utilized as drug nanocarriers, have been discussed.

Kafetzi and Pispas report on the synthesis of hydrophobically modified poly(2-(dimethylamino) ethyl methacrylate)-b-poly(oligo(ethylene glycol) methyl ether methacrylate) diblock copolymers using RAFT polymerization and post-polymerization quaternization of the PDMAEMA block with long alkyl chains in a random fashion [2]. Due to their amphiphilic character the copolymers self-assemble into nanoaggregates in aqueous media in response to pH and temperature and can be used as nanocarriers for indomethacine.

Bogiatzidis and Zoumpoulakis present the preparation of thermoset composites based on commercially available epoxy and unsaturated polyester resins, as well as laboratory synthesized phenolic resin (novolac) filled with construction and demolition (C&D) waste powder of different grain size in the micro-scale, and in the 30–50% *w/w* C&D wastes composition range [3]. The mechanical properties of the composites were investigated.

Pitsikalis and his group present the synthesis of statistical copolymers of N-vinylpyrrolidone and isobornyl methacrylate utilizing traditional free radicals and RAFT polymerization [4]. The reactivities of the monomers were determined in each polymerization scheme. The thermal properties and thermal decomposition of the new copolymers are studied as well.

Sakellariou and coworkers propose the direct functionalization of single-walled carbon nanotubes with diphenylethylene cyclobutene (DPE-CB) end-capped polystyrene chains using a facile, single-step, [4+2] Diels–Alder cycloaddition reaction [5]. Direct evidence for the efficient surface functionalization and the presence of a thin polystyrene film was provided by transmission electron microscopy (TEM) and atomic force microscopy (AFM).

Tsitsilianis, et al. discuss the preparation of mesoporous silica nanoparticles covered by alternative layers (via a layer-by-layer deposition process, LbL) of oppositely charged weak polyelectrolytes [6]. Poly(allylamine hydrochloride) (PAH) and sodium alginate, highly grafted by N-isopropylacrylamide/N-tert-butylacrylamide random copolymers (NaALG-g) were utilized for the LbL assembly. Due to the pH-responsive properties and LCST-type thermoresponse of the NaALG-g polymer, the hybrid nanoparticles exhibit pH and thermo-responsive drug delivery properties. 

Charitidis and coworkers examine the utilization of core–shell super absorbent polymers (SAPs) as triggerable materials in coating binders [7]. SAPs acrylic binders were applied onto acrylonitrile–butadiene–styrene/polycarbonate plastic substrates (at 1 and 5 wt.% SAPs) and facilitated the debonding of the coatings from the plastic substrates by a steam treatment. The study is strongly related to the recycling of plastics.

The contribution by the groups of Vamvakaki and Chatzinikolaidou focuses on the synthesis of novel polyesters with pH-responsive carboxylic groups and alkene functionalities utilizing condensation polymerization and photo-induced thiol-ene click reactions [8]. The novel polyesters show water solubility and biocompatibility, while their crosslinked analogues showed promise as cell culturing substrates and in tissue engineering applications.

Vamvakaki and coworkers also report on the synthesis and properties of novel, linear, main-chain photo- and acid-degradable copolymers based on acylhydrazone bonds by applying step growth copolymerization based synthetic schemes [9]. Photo- and acid-induced degradation of the copolymer in dimethylsulfoxide and water was demonstrated by UV-vis spectroscopy. The authors succeeded in attaching the anticancer drug doxorubicin onto the copolymers forming amphiphilic drug-polymer conjugates which form nanoscale aggregates in aqueous media. UV light and acid degradation of these particles result in the efficient release of the drug cargo.

Paipetis, et al. study the healing properties of urea-formaldehyde (UF) shell-wall microcapsules after the addition of multi-walled carbon nanotubes (MWCNTs), under different concentrations of the filler particles [10]. The addition of MWCNTs resulted in a decrease in the size of microcapsules and an improvement in the electromagnetic interference shielding effectiveness and the mechanical properties of the composites, even at low MWCNT content, without affecting the healing properties of the materials.

Manouras, et al. investigate the antibacterial properties of lightly quaternized poly(2-(dimethylamino)ethyl methacrylate)s which were prepared using alkyl halides of different alkyl chain structures [11]. Quaternization affected the LCST behavior of the copolymers in aqueous media compared to the PDMAEMA precursor in different ways depending on the nature of the quaternizing agent. The copolymers show inhibitory action against *Escherichia coli* and *Staphylococcus aureus* strains.

Tsioptsias, et al. study the tensile strength and thermal stability of isotactic polypropylene (PP) composite drawn fibers reinforced with five different fillers (microtalc, ultrafine talc, wollastonite, attapulgite, and single-wall carbon nanotubes) [12]. The use of ultrafine talc particles results in better mechanical and thermal performance.

Raptis and Karatasos present a theoretical investigation of the interactions of essential oil ingredients, having therapeutic potential, with hyperbranched PEI and PG homopolymers in aqueous media using molecular dynamics simulations [13]. Despite the fact that clustering phenomena are observed for the terpenoids under study, sufficient polymer-drug interaction and association are observed over the whole composition range.

Finally, in their review article, Papagiannopoulos and Sotiropoulos discuss the methods for the preparation of polysaccharide nanogels and microgels as well as their potential utilization in biomedical and food research, mainly as nanocarriers for bioactive substances and emulsion stabilizers [14]. 
